# Surgery

**Published:** 1860-07

**Authors:** S. W. Gross

**Affiliations:** Lecturer on Surgical Anatomy and Operative Surgery, Philadelphia


OR, BI-MONTHLY NOVELTIES IN MEDICAL, PATHOLOGICAL, SURGICAL, OBSTETRICAL, PHYSIOLOGICAL, AND CHEMICAL SCIENCE.

### SURGERY.

BY S. W. GROSS, M.D.,
LECTURER ON SURGICAL ANATOMY AND OPERATIVE SURGERY, PHILADELPHIA.

I.—*On Lithotomy, considered as a Cause of Death. With Remarks on the Present State of the Operation usually called the "Rectangular Operation for the Stone;" and on the best Method of extending the ordinary Incisions in that Operation, for the purpose of extracting Stones of unusually large size.* By Professor A. Buchanan, M.D., University of Glasgow. (Glasgow Medical Journal, April, 1860.)

It is known to most of our readers that the operation of lithotomy by the rectangular staff was first introduced into practice in 1846, by Professor Buchanan, and, with the exception of the Glasgow surgeons, it has not been generally adopted. Indeed, so far as we have been able to learn, it has been performed but twice out of Glasgow—once by Mr. Spence, of Edinburgh, and once by Professor Gross, at the clinic of the Jefferson Medical College, about eighteen months ago. Quite a number of modifications have been made in the rectangular staff, and in the method of holding it; but to perform the operation as described by the author, the instrument must be held in one invariable position, that is, with the groove under the level of the bulb of the urethra, which will permit the knife to penetrate into the bladder by the shortest possible route.

Dr. Buchanan is very sanguine as to diminishing the mortality of lithotomy by his method, and thinks that, when adopted, it will show a success greater than that attained by any other operation. Of upwards of sixty cases, cut by ten different surgeons, the average number of deaths has been but one in twelve; and although founded upon so limited a number of cases, the success shows a great saving of life, which is the more satisfactory, since the operation has undergone many important modifications, as suggested by extended experience, from the date of its introduction. In the six fatal cases death ensued very rapidly, and in five of these the post-mortem appearances were singular, and identically the same. The first fatal case occurred in the practice of the late Dr. Lawrie, the patient being dead in a little more than twenty-four hours. There was found intense ecchymosis of the superior and posterior surfaces of the bladder and of the adjacent parts, and on lifting up the intestines a small quantity of sanguinolent fluid was discovered on each side of the abdominal cavity. The peritoneum showed no signs of inflammation. Four of the other cases were charaterized by the same appearances, which existed in a greater or less degree, and in one the ecchymosis extended along the whole of the rectum and a part of the sigmoid flexure of the colon. The sixth fatal case does not come under this category, but death was caused by mechanical contusion of the bladder and of the perineum. All these patients, therefore, died of injuries inflicted by the operation, from special errors in the mode of cutting.

Four causes render the operation for stone in itself dangerous, when performed upon persons of good general health, and in whom the organs are not unsound, namely—1. Infiltration of urine into the subserous cellular tissue of the peritoneum, and the passage of it through that membrane, by osmosis or simple percolation, into the interior of the peritoneal cavity. 2. Infiltration of urine into the subfacial cellular tissue of the pelvis, while all access to the peritoneal cavity is debarred by the dense fibrous membrane. 3. Mechanical contusion of the bladder and parts adjacent, of which what is called "shock" is the most severe form. 4. Hemorrhage.

1. Infiltration of urine in itself is not necessarily a cause of death, excepting in certain regions, as is well seen in external effusions of this fluid into the perineum. The first form of infiltration had never been recognized as a distinct affection until the rectangular operation came into use, whereas the other form occurs after the lateral operation. The five cases of death, briefly noticed above, come under this head, and in regard to one, in which the ecchymosis had involved the rectum and a portion of the sigmoid flexure of the colon, Dr. Buchanan remarks: "I traced the blood all around the rectum, where that intestine is covered with peritoneum, and beyond the reflection of the peritoneum I found the blood extending downward, between the rectum and bladder, as far as the external wound." The blood had, therefore, traveled from the external wound into the cellular tissue, between the rectum and bladder, as far as the recto-vesical pouch, and, following the course of the peritoneum, had diffused itself over the bladder, the rectum, and the colon. The fluid found in the peritoneal cavity was urine mixed with blood, which, having followed the same route as the latter fluid, entered the abdomen by osmosis, and thus produced rapid death.

2. This cause of death has never been observed after the rectangular method, but should it occur, the patient will not be so seriously affected immediately after the operation, nor will dissolution take place so rapidly as in the first form.

3. Mechanical contusion of the bladder and perineum resulted fatally in one case. The stone was of large size, and the usual incision on the rectangular staff having been made, it was prolonged upward; but this not allowing the extraction of the calculus, the bilateral section of the soft parts and of the prostate was resorted to. Death ensued in four days, from subacute peritonitis.

4. None of the cases perished from this cause; but should the staff be held too high, instead of with the groove under the level of the bulb of the urethra, the spongy portion and bulb will be wounded, and a profuse hemorrhage be the result.

In regard to the best mode of extending the ordinary incisions of the rectangular operation for the purpose of extracting stones of large size, the author advises the upward incision, for by cutting too far laterally or downward, risk is run of urinary infiltration. The upward incision can even be carried beyond the base of the prostate, dividing the bladder to any necessary extent, and this, with the lateral incision first made, will permit the extraction of a voluminous calculus. The mode in which Professor Buchanan performs the incision upward is by means of the *finger-director*, which he thus describes: "It is a metallic cylinder, tapering from the rounded point to the base, open in front, and fitting like the finger of a glove to the index finger, while the handle attached to the back part of it lies flat upon the back of the hand. This tapering cylinder, fitted upon the index finger, enters the wound with the same facility as the index itself; and the part of it at which its further progress is arrested gives an accurate measurement of the circumference of the wound of the prostate. The handle of the instrument being now securely held, while the open side of the instrument is turned upward, the forefinger feels with much nicety the tense fibres stretched over the open groove which require to be divided, and this is done by one or more incisions with a curved bistoury."

[The only case of lithotomy, in which the method of Professor Buchanan has been resorted to in the United States, occurred at the clinic of the Jefferson Medical College, in the hands of Professor Gross, on the 18th of December, 1858. The patient, a young man seventeen years of age, had shown well-marked symptoms of stone for fifteen years, and his general health was bad. Two very large uric acid calculi were removed, and the operation was attended with considerable hemorrhage, all the arteries of the perineum being much enlarged in consequence of the long-continued irritation excited by the presence of the calculi. On the third day the urine came away by the urethra, and the patient was discharged cured at the expiration of three weeks.]

II.—*New Operation for the Removal of Naso-pharyngeal Polyps.* By PROF. B. LANGENBECK. (Deutsche Klinik, No. 48, 1859.)

Since attention has again been directed to the osteogenic properties of the periosteum, through the interesting physiological researches of M. Ollier, surgeons have evinced a growing zeal in applying the facts deduced from his experiments to their various operative procedures on the bony structures, and in a paper on "Osteoplastic Operations," by Professor Langenbeck, of Berlin, we find that this distinguished surgeon has devised a new and ingenious operation for the removal of naso-pharyngeal polyps, showing that reunion in a bone, which has been separated from its osseous connections, can be effected, provided it remains united by periosteum to the parent bone.

The case was that of a young man, eighteen years of age, who was admitted into the surgical clinic of the University of Berlin, on account of two large fibrous polyps which impeded respiration, and occupied the space behind the soft palate. Their large volume did not permit their removal through the nose, or by section of the hard and soft palates, and Professor Langenbeck, therefore, with the view of avoiding great disfigurement, resected the right nasal bone, and the nasal process of the superior maxilla, saving a bridge of periosteum, by which these bones adhered to the surrounding parts, and promoted their union when replaced. The operation was performed in November last, by carrying an incision from the nasal eminence over the right nasal process of the superior maxilla to the wing of the nose. The skin was then dissected up, so as to leave the periosteum intact, and the bones being thus fully exposed, were detached from the wing of the nose. Cutting forceps, carried along the nasal suture as far up as the nasal spine of the frontal bone, separated the right nasal bone from the left, and the base of the nasal process was divided by a transverse cut extending into the maxillary sinus. The bones were then raised by an elevator introduced into the nose, and the nasal bone being separated from the frontal, the bony flap was held up on the forehead, thus allowing the easy extraction of the polyps. The luxated bones, which remained adherent by a flap of periosteum and of mucous membrane an inch in width, were then replaced, and the wound was brought together by silver sutures. On the fifteenth day after the operation the cure seemed to be complete. For the first few days there was some swelling of the soft parts, which disappeared under the use of cold water, and when the patient was dismissed the wound was thoroughly united, and no pain was experienced by pressure upon the resected bones, although there yet existed slight tumefaction over the nasal process of the superior maxilla.

III.—*Ligature of the Femoral Artery for the Cure of Elephantiasis of the Leg and Foot.* By T. L. OGIER, M.D., of Charleston, South Carolina. (Charleston Medical Journal, March, 1860.)

In conformity with the practice of Professor Carnochan, Dr. Ogier ligated the femoral artery of a negro man, twenty-six years of age, for elephantiasis, of enormous size, of the leg and foot, the disease having existed for five years. The vessel was tied without difficulty in the lower portion of Scarpa's triangle; but traumatic fever followed the operation, which was successfully combated by the tincture of veratrum viride. The leg and foot began to decrease in size after the second day, and on the twelfth day, when the ligature came away, the volume was not more than one-half that it was before the operation. On the morning of the fifteenth day secondary hemorrhage occurred, which was controlled by a firm compress, retained for twelve days by means of a bandage. Three months after the ligation of the artery, the affected tissues had almost resumed their natural size, and the patient walked about without experiencing any uneasiness.

[When the reporter was one of the resident physicians of the Philadelphia Hospital, he witnessed the operation of ligation of the femoral artery for elephantiasis of the right leg and foot, on a negro man, thirty-eight years of age. The parts were very voluminous and of dense consistence, and the disease had existed for nine years, being accompanied by constant pain. The artery was tied on the 17th of May, 1857, by Dr. Campbell, then surgeon-in-chief, and the ligature came off on the thirteenth day. No perceptible alteration in size followed the operation, and a year afterwards the condition of the leg and foot was not materially changed.]

IV.—*On the means to be employed in Syncope from Hemorrhage after Surgical Operations.* By M. DEBOUT. (Bulletin Général de Thérapeutique, Feb., 1860, and Lancet, May 12, 1860.)

In a paper published in 1859, M. Debout showed the good effects which he had obtained in a case of syncope following parturient hemorrhage, from the use of the heated steel button and injections of wine, and he thinks that transfusion of blood is indicated when these means fail. In the fainting resulting from surgical hermorrhage the treatment must be different, and the author concludes his valuable paper with the following propositions:—

1. Syncope following traumatic hemorrhage is a more serious symptom than the fainting resulting from uterine hemorrhage, both on account of the nature of the blood lost, and its less rapid escape; it may, therefore, be affirmed that the means found successful in the latter complication are not, as a consequence, likely to be efficient in the former.

2. As syncope, in traumatic hemorrhage, is the result of want of stimulus to the nervous centres, the remedy will consist in putting the patient upon his back, and in lessening the extent of the circulation by abdominal pressure.

3. As we are to be especially anxious for the energy and duration of our therapeutic interference, we should add to the physical action of the means above alluded to the stimulation of the heated steel knob, rapidly carried over various parts of the cutaneous surface, and assist this operation by enemata of wine. This syncope is so perilous that all the above measures combined will never be too powerful. The analysis of a small number of cases proves that transfusion of blood affords but short relief in these cases.

V.—*Secondary Amputations after Gunshot Wounds.* By M. Jules Roux. (Journal de Progrès, 27th April, 1860.)

On the 24th of April of the present year, M. Roux, of Toulon, presented to the Paris Academy of Medicine a very interesting paper on secondary amputations in gunshot wounds attended with fracture of the bone, and discussed more especially the important subjects of osteomyelitis, and the substitution of consecutive disarticulation for amputation in the continuity of a limb. As one of the surgeons of the Marine Hospital of Toulon, the author has had ample opportunity of studying this class of affections, and he advises, when the osteomyelitis has existed for some time, to disarticulate a limb above the point of injury, thus getting rid of all the disease. His success in these cases has been remarkable, for we find that of twenty-two operations all were cured, and these include four coxo-femoral and thirteen shoulder disarticulations.

The great majority of surgeons will not be disposed to agree with all the opinions of the author in regard to osteomyelitis, for it is certainly not of so frequent occurrence as he states, and when it does exist, is often spontaneously cured. Moreover, surgeons in these cases have amputated limbs in their continuity and saved their patients, notwithstanding they have found the bone and marrow seriously affected. The conclusions of M. Roux, deduced from numerous cases, are the following:—

1. Osteomyelitis is inevitable after gunshot wounds, but it is most frequently cured.

2. It attacks more or less promptly the whole of the bone, and is here a fixed pathological fact.

3. Secondary amputation or resection hazarding the non-removal of all the disease, leave a part of the affected bone.

4. We may attribute to these partial operations upon the bone primarily affected, the unsuccessful results which lead most often to the death of the patient, and which are perhaps the principal cause of the want of success of secondary amputations.

5. If in from six months to a year after the receipt of a gunshot wound a cure has not taken place, and an operation is indispensable, it will be necessary, in the majority of cases, if not in all, to disarticulate the diseased bone and give up resection and amputation in the continuity.

In an interesting discussion which followed the reading of the paper, M. Larrey made many valuable remarks, showing the results of his large experience in this affection, and regretted that M. Roux had not sufficiently pointed out the anatomical characters of the three stages of the disease, or those of hyperæmia, softening, and suppuration. He thought the following propositions of M. Roux too absolute, namely—First, that in the first period, or that of hyperæmia, the osseous wound must always suppurate; and, second, that the stage of softening coincides with a special pathological condition of the marrow, which most frequently necessitates the removal of the limb. In regard to the second stage, it is not always possible to discriminate between this and the first, and there consequently exists a favorable chance for resolution without sacrificing the member. M. Larrey does not accept, without reserve, the conclusion of M. Roux, that in the third stage, or that of suppuration, amputation is absolutely necessary, since suppuration of the medullary canal may be cured without necrosis being produced, and even if the bone dies, it may be discharged as a sequestrum, which sometimes represents the whole of the shaft. Should, however, extraction of the dead bone or resection of the articulation be impracticable or insufficient for a cure, secondary amputation will be required.

M. Larrey submitted to the Academy the following modifications, which he desired to make in the conclusions of M. Roux:—

1. Osteomyelitis, after gunshot wounds, is more frequent than it has heretofore been considered; but it is not inevitable, and is most frequently cured.

2. It may be limited to one point of the bone, or even attack the whole more or less quickly.

3. It must be subjected immediately to all the rational modes of treatment, when it is susceptible of frequent and even of spontaneous cure.

4. It sometimes requires secondary resection or amputation, at one time in the continuity of the limb, at another, and occasionally preferably, in the contiguity.

5. It can explain, in certain cases, the want of success of partial operations, or operations practiced on bones attacked by this inflammation.

6. It demonstrates, finally, the fitness as well as the success of disarticulations in many cases; but it cannot justify, at least in our eyes, the proposal, much too exclusive for surgeons, of renouncing articular resection and amputation in the continuity of the limb.

7. The simple proposition brought forward by M. Roux, in spite of his great authority in the practice of the art, in spite of the extreme interest and unexpected novelty of his observations, in spite even of the remarkable success of his exceptional practice, in spite, finally, of the serious attention which his researches will awaken in every surgeon, this simple proposition will with difficulty become a precept justified by experience and sanctioned by the Academy.

VI.—*On the Treatment of Certain Diseased Conditions of the Hip-Joint by Complete and Partial Excision of the Articulation.* By Mr. PRICE. (Lancet, April 28, 1860.)

Mr. Price read a paper on the above subject before the Medical Society of London, stating the results of a practical and widely extended experience. At the Infirmary for Scrofulous Children, at Margate, there had come under his observation one hundred and fifty-three cases of disease of different joints, seventy-six of which related to the hip. Of these, thirteen were cured with useful limbs; ten died; forty-one were greatly benefited; eight were slightly improved; in two a portion of the diseased joint was resected with much improvement; and in two the result of treatment could not be ascertained. The statistics of excision of the hip-joint are more fully treated of by Mr. Price than by any author with whom we are acquainted, and we find that he has collected sixty-seven cases, in fifty-nine of which he had authenticated particulars. After entering upon the history of the operation, the author discussed the subject in relation to the special condition of disease for which the procedure had been instituted, and divided the consideration of the subject into the following heads:—

1. Where the diseased head of the femur was dislocated from its acetabular connections, and no disease of the pelvis apparently existed. Sixteen cases were reported with this condition, three of which were fatal, one from erysipelas on the fifteenth day, and two from general debility and tuberculosis of the lungs. In ten instances the condition of the patients was greatly improved, and useful limbs resulted from the operation, and three cases were successful as far as the operation was concerned.

2. Where the diseased head of the femur was dislocated from the acetabulum, and more or less disease of the acetabular portion of the pelvis existed. The operation in these cases was resorted to rather as a palliative measure, the disease being too far advanced to hold out much hope of success. Of eighteen instances, only six were positively cured. Of the twelve unsuccessful cases, the direct effects of the operation led to death in only five, one fatal result being owing to erysipelas. The remaining six cases were more or less benefited by the operation for a certain length of time, although death ultimately ensued, either from diseased kidneys, tuberculosis, disease of the heart, or erysipelas.

3. Where the diseased action was more or less confined to the synovial and cartilaginous structures of the articulation, and if the bony portions of the joint were involved, such inclusion was not of the nature of caries and necrosis. Five cases were reported with this condition, of which three were completely cured, two of these having been operated upon by the author. Of the two fatal cases, one died from irritation and debility on the twentieth day; the other lived for fifteen months, and succumbed from other causes than disease of the hip.

4. Where the integrity of the joint was more or less destroyed, either femoral or pelvic, or both, although no dislocation or rupture of the capsular ligament had occurred. When the local parts were thus involved, fourteen cases were subjected to the operation, with the result of eleven recoveries, with more or less useful limbs, and three deaths, the time varying from three days to three months.

5. Under this head are included those cases, five in number, of which Mr. Price had no exact knowledge. Recovery, however, took place in three, and death in two.

Of these fifty-nine cases, fifty-three of which were operated upon by British surgeons, thirty-three recovered, their general condition being much improved, and with useful limbs. Eleven were so far successful that they lived for periods varying from three months to two years, their general condition, both locally and constitutionally, being greatly benefited. Fourteen cases succumbed from the effects of the operation, and in one Mr. Price was unable to obtain the result.

VII.—*Severe Burn followed by Exfoliation of the whole upper portion of the Skull.* By HARVEY J. PHILIPOT, M.D., M.R.C.S.L. (British-American Journal, May, 1860.)

On the 27th of October, 1857, an Irish woman was seized with a fit and fell into an open wood-fire, inflicting a severe burn upon the neck, face, and scalp, and denuding the upper two-thirds of the frontal and parietal bones, which were charred by the flames. A severe shock to the nervous system was the result, but readily yielded to treatment, and, under the use of a slightly stimulating lotion, a large slough was thrown off in the course of several weeks, leaving a granulating surface on the free margin of the scalp surrounding the exposed bones. When the patient was again seen by Dr. Philipot, ten months after the accident, he found her head bound up with greased rags, and she informed him that while applying the lotion, on the 15th of August, 1858, she felt the bone move, and, by using a little force, she succeeded in removing the top of her head. The bone measured five inches and three-fourths in its longitudinal diameter, and four inches and a half transversely, and its internal table had almost entirely disappeared. The whole superior surface of the cerebrum was exposed, but covered by its membranes, and resembled a large fleshy, pulsating tumor. At the time of reporting the case, nearly two years and a half after the accident, the patient seemed to suffer but little inconvenience from the loss.

VIII.—*On the Treatment of Cystic Goitre.* By PROFESSOR V. BRUNS, of Tübingen. (Deutsche Klinik, und Prager Vierteljahrschrift, 1860, i.)

For the cure of cystic tumors of the thyroid gland, Professor v. Bruns recommends the injection of the sac with from one to two drachms of the undiluted tincture of iodine, introduced through a delicate canula, which closely fits the nozzle of a syringe. The small puncture made by the trocar is closed by adhesive strips and a compress of lint, and the patient is kept at rest in the recumbent posture. On the first or second day after the operation, symptoms of inflammation manifest themselves, and the cyst generally fills up to its former volume. The general health of the patient remains perfectly good, or there is but slight febrile disturbance, which abates in the course of two or three days. After this the cyst gradually grows smaller, and, with one exception, a complete cure without a relapse was the result of this treatment in every case in which it was employed.

IX.—*Cure of a Gastric Fistule by a Plastic Operation.* By PROFESSOR MIDDELDORPF. (Gazette Hebdomadaire, vol. vii., No. 7, 1860.)

The only authentic case of a cure of a gastric fistule by a plastic operation is reported in a memoir, entitled "De Fistulis Ventriculi Externis et Chirurgica Earum Sanatione, Accedente Historia Fistulæ Arte Chirurgorum Plastica Prospere Curatæ," by Professor Middeldorpf, of Breslau, who relates a case of this nature, and gives a complete historical résumé of the subject. The patient was a woman, forty-seven years, of age, and the disease, which dated from her youth, had arisen from a blow on the left hypochondrium. From her twentieth to her thirty-second year, she experienced considerable pain in this region, when a new blow caused a tumor to appear on a level with the costal cartilages, which increased in size for eight years, and resulted in an abscess. This opening, a fistule was the result, for which palliative treatment by means of bandages was first instituted, but not succeeding, a plastic operation was determined upon and performed in 1857. The wound was thoroughly united at the end of the thirteenth day, and remained firm until the sixty-ninth day, when pain set in, and a small opening appeared at the original site of the fistule, which finally became closed under the use of nitrate of silver.

